# The Effects of Composite Resin Types and Debonding Pliers on the Amount of Adhesive Remnants and Enamel Damages: A Stereomicroscopic Evaluation

**DOI:** 10.5681/joddd.2013.032

**Published:** 2013-12-18

**Authors:** Parisa Salehi, Hamidreza Pakshir, Navid Naseri, Tahereh Baherimoghaddam

**Affiliations:** ^1^Orthodontic Research Center, Shiraz University of Medical Sciences, Shiraz, Iran; ^2^Associate Professor, Department of Orthodontics, Faculty of Dentistry, Shiraz University of Medical Sciences, Shiraz, Iran; ^3^Professor, Department of Orthodontics, Faculty of Dentistry, Shiraz University of Medical Sciences, Shiraz, Iran; ^4^Specialist in Orthodontics, Department of Orthodontics, Faculty of Dentistry, Shiraz University of Medical Sciences, Shiraz, Iran; ^5^Assistant Professor, Department of Orthodontics, Faculty of Dentistry, Yasuj University of Medical Sciences, Yasuj, Iran

**Keywords:** Dental debonding, dental enamel, orthodontic adhesive, orthodontic appliance

## Abstract

***Background and aims.*** This *in vitro *study was designed to evaluate the effect of composite resin types and orthodontic debonding pliers on the amount of adhesive remnants and enamel damages using a novel method of measurement.

***Materials and methods.*** 120 extracted human premolars were randomly divided into four groups (n = 30). The standard edgewise metallic brackets were bonded to the teeth with light-cured composite resin “Transbond XT” in the first and second groups, and No-mix composite resin “Unite” in the third and fourth groups. Bracket debonding was carried out with Lift Off Debonding Instrument “LODI” (3M Unitek) in the first and third groups, and with Bracket Removing Pliers (Dentaurum) in the second and forth groups. yStereomicroscopic evaluation was performed by unbiased sterelogy. All teeth were evaluated for the amount of adhesive remnants and the number and length of enamel cracks. The obtained data on each step was analyzed by two-way ANOVA, chi-square, Wilcoxon, and Kruskal-Wallis.

***Results.*** Teeth in group 4 had the lowest adhesive remnants on the enamel surface (p < 0.01); and the highest increase in the number of enamel cracks (p < 0.01) among the groups. In addition, groups bonded with Unite showed the highest increase in the length of enamel cracks.

***Conclusion.*** Dentaurum debonding pliers with sheer–peel force, when used with the Unite adhesive (group 4), decreased the amount of adhesive remnants on the enamel surface while it increased enamel damages.

## Introduction

Bond strength plays an important role in the orthodontic treatment. Bond strength should be high enough to prevent debonding of brackets during treatment and in the mean time, it should be low enough to minimize enamel damages during bracket removal.^1,2^Excessive bond strength can create failure site at the enamel–adhesive interface, and increase the risk of enamel damages.^3,4^

At the end of fixed orthodontic treatments, debonding procedures result in 30-40 μm enamel surface missing.^5,6^ Debonding procedures have a high potential for enamel damage as well as dental pulp irritation, which is influenced by the site of bond failure.^7,8^Debonding methods that contribute to cleaned enamel surface and bond failure site in enamel–adhesive interface will increase the risk of enamel damages and fractures. Enamel damages, such as enamel cracks, fractures, splits, and even cusp fractures, are irreversible and permanent.^8-11^

On the other hand, when a great amount of adhesive remains on the enamel surface after bond failure at the bracket–adhesive interface, more finishing procedures are required to clean the enamel surface. This will increase enamel damages such as scratch, gouging and fissures.^3,4^ Thus, safe removal of brackets, without enamel damages, is as important as bracket bonding.^12^

According to pioneer studies, enamel damages are influenced by debonding methods and various methods have been suggested to provide proper control during debonding.^14-16^ Lift Off Debonding Instrument (LODI) with tensile force, and Bracket Removing Pliers with shear–peel force via squeezing bracket wings can result in bond failure at the bracket–adhesive interface with less enamel damages. However, ligature cutter by applying shear force at bracket base, and Bracket Removing Pliers, including How Pliers and Weingart, by applying pressure force at the bracket base can lead to bond failure at enamel–adhesive interface with more enamel damages.^7,11,15-17^

In addition to the debonding methods, materials that are used in debonding process have different molecular structures and mechanical and physical properties which can play an important role in enamel damages during brackets removal.^18-20^ In some studies, the type of material used had a significant effect on the bond failure site,^16,21,22^ while in others no significant difference was reported.^15^

To the best of our knowledge, few studies have investigated the effects of composite resin types and debonding methods on the bond failure site as well as enamel damages. The present study aimed to compare the effect of two different debonding methods on Adhesive Remnants Index (ARI) and enamel damage, utilizing LODI pliers and Dentaurum Debonding Pliers with two types of composite (Transbond XT and Unite).

## Materials and Methods

### Teeth 

In this *in vitro* study, 120 human permanent premolar teeth, without any caries or visible defects and freshly extracted for orthodontic reasons were selected and used. Before conducting the study, extracted teeth were rinsed with water and stored in an aqueous solution of 0.1% thymol (weight/volume) for 24 hours. The teeth were then kept in distilled water for 6 months at room temperature. The teeth were randomly divided into four groups of each 30 specimens. Prior to the experiments, the buccal surfaces of all teeth were cleaned and polished with a rubber cup and non-fluoridated pumice using a low-speed hand piece for 10 seconds. The teeth were put in acrylic cylinders from their root.

### Bonding 

In this study, 0.018 inch standard Dyna-Lock premolar brackets (3M Unitek, Monrovia, California, USA) were used. The brackets were bonded to the enamel surface with two types of adhesive, according to the manufacturer’s instructions: light-cured composite “Transbond XT” (3M Unitek, Monrovia, California, USA) in the first and second groups and No-Mix self-cured composite Unite (3M Unitek, Monrovia, California, USA) in the third and fourth groups.

In the first and third groups, LODI pliers (3M Unitek, Code 761_444) were used. LODI was placed by inserting its hanger to the upper left bracket and simultaneously resting the pliers on the tooth. Compression of the pliers caused tensile force ( Figure 1a).


Figure 1. (a) Applied tensile force by LODI pliers for bracket debonding. (b) Applied shear–peel force by Dentau-rum Debonding pliers via squeezing bracket wings and its base distortion.

**a:**
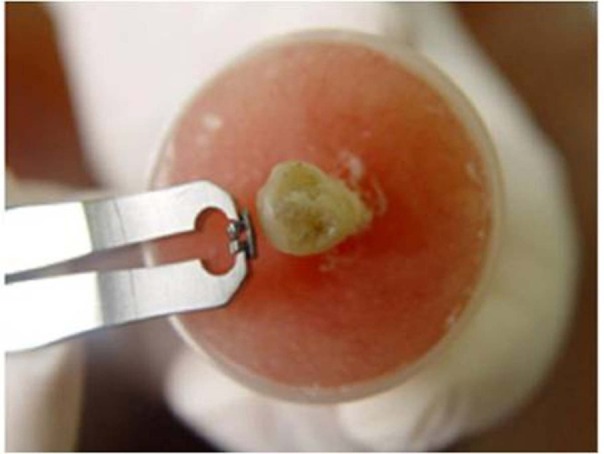

**b:**
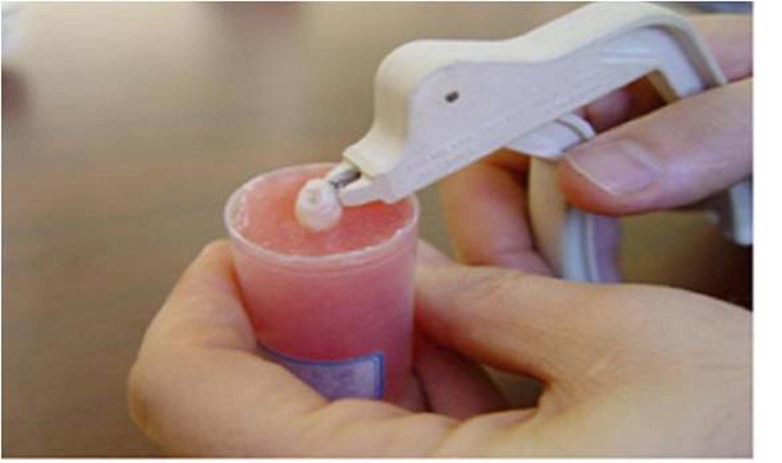

In the second and fourth groups, brackets were removed through applying shear–peel force by means of bracket removing pliers (Dentaurum, Code 00-003) to squeeze bracket wings and to distort the bracket base, respectively (Figure 1b).

### Microscopic Evaluation

The number and the length of enamel cracks were evaluated using a stereomicroscope before bonding and after debonding for each specimen. To this end, a stereomicroscope (Nikon, SMZ745T, Japan) was employed at ×38 magnification with a connected digital camera (Nikon, SMZ745T, Japan) and a computer software (Stereolith Version 1, designed at Histomorphometry and Stereology Research Center, Shiraz University of Medical Sciences, Shiraz, Iran).^23-25^

To determine the amount of adhesive remnants, the total enamel surface specified to the bonding area was divided into 96 points, and each point stood for one unit with a specific calculable area which was determined to be 0.126 mm^2^using the following mathematical formula:^23,24^

TotalArea=∑PX(a/p)

$$(a/p)=\frac{\Delta X\times \Delta Y}{m^{2}}$$

(Σ=sum of points, a= area, p = point, m = magnification coefficient)

The bonding surface area was calculated at 12.096 mm^2^, which was equal to the bracket base area. Thus, the area of adhesive remnants (in mm^2^) was calculated through counting the points area where adhesive was left on the enamel surface.^26,27^ The percent of the adhesive remnants area on the enamel surface was then determined via dividing the adhesive remnants area to the bonding surface area (12.096 mm^2^).

The same procedure was applied to measure the amount of adhesive remnants on the bracket surface and its percentage.

Then, according to the 4-point scale of ARI, described by Artun and Bergland,^28^ ARI scores were graded as follows:

0: No adhesive left on the tooth surface

1: Less than half of the adhesive left on the tooth surface

2: More than half of the adhesive left on tooth surface

3: All adhesive left on the tooth surface

For better determination of the number and the length of enamel cracks and their direction, each tooth was rotated 360 degrees around the center point of the buccal surface and simultaneously the light with a 45 degree angle; because the cracks would not be detected if the direction of enamel crack and the light were the same. The length of enamel cracks was measured via Stereolith (version 1) in µm and then converted to millimeter. The structural design of the buccal tooth surface, length, direction, and the number of cracks were recorded on paper by one examiner. One number was assigned to each crack. This procedure was repeated after debonding, the length and number of cracks were recorded for comparison. All teeth were evaluated by a second senior examiner for enamel damages. Inter-examiner reproducibility gave a Cohen’s Kappa test of 0.92. Data recorded by first examiner were analyzed by statistical test.

### Statistical Analysis Test 

To assess the amount of adhesive remnants either on the enamel surface (in mm^2^ and percentage), two-way analysis of variance (ANOVA) was employed. All treatment combination means were compared using the* post hoc* Tukey’s multiple comparison tests. The chi-square test was performed to compare the ARI score among the groups.

To determine significant differences in the number and length of enamel cracks before bonding among the groups, Kruskal-Wallis test was used. To determine significant increasing in the number and length of enamel cracks before and after debonding in each group, Wilcoxon and paired sample tests were employed, respectively.

To compare the number and length of enamel cracks increased before and after debonding among the four groups, two-way analysis of variance (ANOVA) was employed. Four groups were compared using the *post hoc* Tukey’s multiple comparison tests.

## Results

The results of two-way ANOVA used to determine significant differences in adhesive remnants on the enamel surface for adhesive type (P < 0.004) and debonding instrument (P < 0.001) are shown in Table 1. A significant interaction between adhesive type and debonding instrument was observed (P < 0.010). The results of Tukey’s multiple comparison tests showed group 4 (Unite composite and Debonding Dentaurum Pliers) to have the least adhesive remnants on the enamel surface when compared to the other groups (P < 0.01; Table 2). The percentage of total adhesive which remained on both enamel and bracket surfaces in most specimens was higher than 100%, implying that an intra-composite fracture or cohesive failure of composite had occurred; group 4 had the least cohesive failure. Frequency distribution of ARI scores and chi-square comparison of the groups are presented in Table 3. No significant difference in the number and length of enamel cracks was found among four groups before bonding (Table 4). The results of two-way ANOVA showed significant differences in the number of enamel cracks before and after debonding with regards to adhesive type (P < 0.04) and debonding instruments (P < 0.001). The result of two-way ANOVA also revealed significant differences in the length of enamel cracks before and after debonding with regard to adhesive type (P < 0.01).

**Table 1:** Two-way analysis of adhesive remnant on the enamel surface with respect to the adhesive and debonding instrument used

Source of variance	Sum of square	Mean square	Df	F ratio	Significance
Adhesive type	132.054	132.054	3	6.9	0.004
Debonding instrument	371.882	371.882	3	36.9	0.001
Adhesive type × debonding instrument	81.824	81.824	1	0.9	0.010
Error	804.738				
Corrected total	1423.379				

**Table 2:** Mean and standard deviation of adhesive remnants on the enamel and bracket surface among the four study groups

Group		Mean ± SD	P-value	Mean ± SD	P-value
		Enamel		Bracket	
1	Transbond XT and LODI	50.13±32.90 A	P<0.001^**^	74.44±28.40 A	P<0.001^**^
2	Transbond XT and Dentaurum pliers	53.85±31.74 A		70.38±22.23 A	
3	Unite and LODI	53.43±39.30 A		68.64±25.09 A	
4	Unite and Dentaurum pliers	28.76±26.22 B		88.78±14.49 B	
^*^p<.05; ^**^p<.001

**Table 3:** Frequency distribution of the Adhesive Remnant Index (ARI) scores and the chi-square comparison of the four study groups

Test groups		ARI SCORE	
	0	1	2	3	N	P-value
Group 1 (Transbond XT & LODI)	0	17	10	3	30	0.004 ^*^
Group 2 (Transbond XT & Dentaurum)	0	15	13	2	30	
Group 3 Unite & LODI	3	10	13	4	30	
Group 4 Unite & Dentaurum	1	26	2	1	30	
ARI Score: 0, No adhesive left on the tooth surface; 1, Less than half of the adhesive left on the tooth surface ; 2, More than half of the adhesive left on tooth surface; 3, All adhesive left on the tooth surface

**Table 4:** Number and length of enamel cracks before and after debonding in each study group

Groups	The number of cracks	The length of cracks
	Before bonding	After debonding		Before bonding	After debonding
	Mean ± SD	P-value	Mean ± SD	P-value
1	2.03±1.27	4.30±2.30	<0.001^**^	3.54±2.22	10.65±5.39	<0.001^**^
2	2.17±1.04	4.48±2.06	<0.001^**^	4.50±2.50	11.53±6.25	<0.001^**^
3	2.52±1.54	5.17±2.92	<0.001^**^	4.88±3.24	13.78±7.06	<0.001^**^
4	2.07±1.15	6.14±2.77	<0.001^**^	3.39±1.83	13.72±7.43	<0.001^**^
^*^p<0.05;^**^p<0.001

The results of Tukey’s multiple comparison tests showed that group 4 had maximum number of enamel cracks after debonding (Table 4) and group 3 & 4 which bonded with Unite adhesive showed the maximum increasing in the length of enamel cracks (Table 5).

**Table 5:** The differences in the number and length of enamel cracks after debonding among the four groups using two-way analysis of variance (ANOVA) and *post hoc *Tukey’s multiple comparison tests

Groups	The number of cracks	The length of cracks
	Mean ± SD	P-value	Mean ± SD	P–value
1	2.27±1.87 A	<0.001^**^	7.11±4.64 A	<0.001^**^
2	2.31±1.83 A		7.02±5.45 A	
3	2.65±2.39 A		8.90±5.96 B	
4	4.07±2.50 B		10.32±6.95 B	
^*^p<0.05;^**^p<0.001

## Discussion

Orthodontic bracket debonding has been shown to be a detrimental process for enamel surface.^7,8^Debonding method, adhesive type, and finishing instruments are among the factors that determine the enamel damages after deboning.^18-20^ Debonding is inevitable after fixed orthodontic treatment, and therefore, selecting a proper technique can reduce enamel damages. In line with previous studies, the results of the present study indicate that increase in the number of enamel cracks and the amount of adhesive remnants after debonding are dependent upon the type of adhesive resin and debonding method used.^13^ Although the type of adhesive had a significant effect on increasing the length of enamel cracks, no significant difference was found in the length of enamel cracks after debonding between the two types of debonding instruments used in this study.

The occurrence of enamel fracture depends on several factors such as the patients’ age and the composition of the enamel surface, especially fluoride changes. Thus, extracted teeth from younger individuals were used in the present study to avoid high risk of enamel cracks and fractures.^29^ No significant differences were observed among the four study groups in the number and length of enamel cracks before bonding, and therefore, the significant differences between groups after debonding can be considered a result of debonding procedures.

In this study, two types of composite were used, namely Unite (No-mix type) and Transbond XT (light-cured type). Unite composite is commonly used by orthodontists, and according to Willems et al,^30^ No-mix adhesive has the greatest bond strength among all adhesives. LODI pliers with tensile force and Debonding Dentaurum pliers with shear–peel force, via squeezing bracket wings, were employed during debonding procedures as pioneer research indicated that these devices were safer and more reliable than other methods and bracket removal devices.^15,16,31^

The lower ARI score is accompanied by a higher incidence of failure at enamel–adhesive interface. The greater the probability of failure at enamel–adhesive interface, the greater the damages on the enamel surface. Group 4 (Unite composite with Debonding Dentaurum Pliers) showed a significantly increased ARI score of 1, indicating the highest increase in the number of enamel cracks. Therefore, the length of enamel cracks was not seen to be correlated with ARI score.

Group 4 had the least amount of adhesive remnants on the enamel surface and the highest increase in the number of enamel cracks among the four groups studied. It is assumed that the applied shear–peel force decreases the amount of adhesive remnants on the enamel surface to a significantly higher extent compared with tensile force, which increases enamel damages.

In group 4, most fractures occurred at enamel–adhesive interface. Changing the type of adhesive and the debonding instrument seemed to transfer the site of bond failure to bracket–adhesive interface or cohesive failure (intra-adhesive). Katona^7^ believes that the type of applied force and the debonding device are important features which determine the site of the bond failure.^7^ For example, applying shear–peel forces, compared to tensile forces, can alter the bond failure site, and applying tensile forces on a joint, compared to shear forces, leads to failure in the enamel site. Applying tensile force by LODI pliers resulted in bracket–adhesive interface fracture in groups 3 and 4, and by changing the type of force to shear–peel force (Dentaurum pliers), the bond failure site changed to enamel–adhesive interface.

Bennette et al^11^ believe that bond failure in adhesive–bracket interface results in less enamel damages. In addition, applying force on the bracket base and adhesive area leads to stress concentration on the enamel surface and bond fracture will occur in adhesive–enamel interface, and this is detrimental.^11^Bishara et al^32^ made mention of bond fracture in enamel–adhesive interface as a detrimental factor which damages the enamel surface.

The length of enamel cracks increased when Unite was employed, with no significant differences with regards to debonding instruments used, probably due to the nature of filled adhesive like Concise composite.^33^In contrast to the findings of the present study, Knosel et al^14^ found no significant differences between two types of adhesive (composite adhesive systems and glass ionomeric cement) with regards to the enamel damages after debonding. They found the highest and the least amounts of enamel damages with the application of side cutter and impulse debonding, respectively. LODI caused more enamel damages compared to bracket removal pliers; and this difference was attributed to the adhesive types used in their study and the method applied for the evaluation of enamel damages.^14^

Howell and Weeks^34^ used three types of resin, namely Concise (heavy filled), System1 (lightly filled), and Super Bond Orthomite (unfilled). System1, in comparison with Concise and Super Bond, created a more acceptable surface with fine cracks, but the difference was not significant.^34^

With regards to the evaluation of the enamel surface after debonding in previous studies, teeth were kept immovable below the microscope on one end, and the light was emitted from the other end. In the present study, rotation around the buccal surface showed that some enamel cracks or their directions can only be detected if the teeth are not fixed during evaluation. Enamel cracks can be identified when the direction of cracks and the light is perpendicular.

The following conclusions can be drawn for the present study:

1. The application of Dentaurum Debonding Pliers when Unite adhesive was used, compared to the other groups, led to bond failure site at enamel–adhesive interface and the highest enamel damages in the debonding process.
2. During the debonding process, the application of Dentaurum Debonding Pliers especially with Unite adhesive resulted in a lesser amount of adhesive remnants on the enamel surface and a higher number of enamel cracks compared with LODI.
3. During the debonding process, Unite caused a significant increase in the length of enamel cracks in comparison with Transbond XT. No significant differences were found in the length of enamel crack caused by Dentaurum Debonding Pliers and LODI.

##  Acknowledgments

The authors would like to thank the vice-chancellor for research at Shiraz University of Medical Science for supporting this study (Grant #1033). This article is based on the thesis by Dr. Navid Naseri. The authors would like to thank Dr. Mehrdad Vosoghi of Dental Research Development Center, of the School of Dentistry for the statistical analysis.
